# An overview of recent technological developments in bovine genomics

**DOI:** 10.1016/j.vas.2024.100382

**Published:** 2024-07-23

**Authors:** Navid Ghavi Hossein-Zadeh

**Affiliations:** Department of Animal Science, Faculty of Agricultural Sciences, University of Guilan, Rasht, 41635-1314, Iran

**Keywords:** Cattle, Genome editing, Genome-wide association studies, Genomic selection, Machine learning, Single nucleotide polymorphism, Whole genome sequencing

## Abstract

Cattle are regarded as highly valuable animals because of their milk, beef, dung, fur, and ability to draft. The scientific community has tried a number of strategies to improve the genetic makeup of bovine germplasm. To ensure higher returns for the dairy and beef industries, researchers face their greatest challenge in improving commercially important traits. One of the biggest developments in the last few decades in the creation of instruments for cattle genetic improvement is the discovery of the genome. Breeding livestock is being revolutionized by genomic selection made possible by the availability of medium- and high-density single nucleotide polymorphism (SNP) arrays coupled with sophisticated statistical techniques. It is becoming easier to access high-dimensional genomic data in cattle. Continuously declining genotyping costs and an increase in services that use genomic data to increase return on investment have both made a significant contribution to this. The field of genomics has come a long way thanks to groundbreaking discoveries such as radiation-hybrid mapping, in situ hybridization, synteny analysis, somatic cell genetics, cytogenetic maps, molecular markers, association studies for quantitative trait loci, high-throughput SNP genotyping, whole-genome shotgun sequencing to whole-genome mapping, and genome editing. These advancements have had a significant positive impact on the field of cattle genomics. This manuscript aimed to review recent advances in genomic technologies for cattle breeding and future prospects in this field.

**Abbreviations**
SNP: Single nucleotide polymorphism
USDA: United States department of agriculture
QTL-MAS: Quantitative trait loci-marker aided selection
QTN: Quantitative trait nucleotides
WGS: Whole genome shotgun
BAC: Bacterial artificial chromosome
BovineLRC: Bovine long read consortium
NGS: Next generation sequencing
ONT: Oxford nanopore sequencing
PacBio: Pacific biosciences
PCR: Polymerase chain reaction
SMRT: Single-molecule real-time
CNVs: Copy number variations
MARC: Meat animal research center
EST: Expressed sequence tag
CSIRO: Commonwealth scientific and industrial research organization
GWAS: Genome-wide association studies
GEBV: Genomic estimated breeding value
GBLUP: Genomic best linear unbiased prediction
EBVs: Estimated breeding values
Debv: De-regressed estimated breeding value
ssGBLUP: Single-step GBLUP methodology
MCMC: Monte Carlo markov chain
ML: Machine learning
NLP: Natural language processing
IoT: Internet of things
RKHS: Reproducing kernel Hilbert spaces
SVM: Support vector machines
MLP: Multilayer perceptron
RNN: Recurrent neural networks
CNN: Convolutional neural networks
InDels: Insertion-deletions
LD: Linkage disequilibrium
PAVs: Presence/absence variations
MR: Mendelian randomization
mGWAS: Metabolome-based GWAS
PWAS: Proteome-wide association study
TWAS: Transcriptome-wide association study
MWAS: Metagenome-wide association study
EWAS: Epigenome-wide association study
HMM: Hidden Markov models
OSW: Overlapping sliding windows
GnEd: Genome editing
DSB: Double strand breaks
ZFNs: Zinc finger nucleases
TALENs: Transcription activator-like endonucleases
FDA: U.S. food and drug administration
NHEJ: Non-homologous end-joining
SCNT: Somatic cell nuclear transfer
MSTN: Myostatin

## 1 Introduction

It is projected that by 2050, there will be a 70 % increase in global demand for food products originating from animals. The adoption of cutting-edge technologies will be necessary to meet this demand in a way that has the least negative environmental impact. Genetically improved livestock techniques are anticipated to play a significant role in this (Georges et al., 2019). It has long been known that cattle are incredibly valuable livestock because of their milk, beef, manure, hide, and draft power. Sustainable animal husbandry has been and will continue to be significantly influenced by the global genetic improvement of cattle. A structured breeding program with a clear breeding objective is the best way to maximize the impact of various reproductive and molecular biotechnologies used in the genetic improvement of cattle. These biotechnologies work together to maximize the rate of genetic progress (Mueller and Van Eenennaam, 2022). The scientific community has taken different approaches to improving the genetic makeup of the cattle germplasm. Through the use of genomic selection in conjunction with assisted reproductive technologies, livestock genetic improvement programs—which started with selective breeding using statistical prediction methods like estimated breeding values and, more recently, genomic selection —have made it possible to more accurately select and heavily utilize genetically superior parents for the next generation, accelerating rates of genetic gain (Mueller and Van Eenennaam, 2022).

In order to increase the frequency of advantageous alleles and decrease the prevalence of harmful alleles, genomics research on livestock has aimed to correlate variations in genome sequence with phenotypes that impact traits that are significant to animal health, welfare, productivity, profitability, and sustainability. The United States Department of Agriculture (USDA) recently invested up to $40 million a year in the "Agricultural Genomes to Phenomes Initiative" (Tuggle et al., 2022) and established the international Functional Annotation of Animal Genomes "FAANG to Fork" initiative (Clark et al., 2020), which is a project that is similar to the human ENCODE project (ENCODE Project Consortium, 2004; Smith et al., 2023). These developments demonstrate the significance of this research.

Groundbreaking discoveries such as cytogenetic maps, synteny analysis, somatic cell genetics, in situ hybridization, radiation-hybrid maps, and the construction of high-resolution comparative mapping, molecular markers (based on hybridization and polymerase chain reaction), rudimentary genome maps, association studies for quantitative trait loci-marker aided selection (QTL-MAS) [with economically important traits], and whole genome shotgun sequencing to whole genomic mapping have significantly advanced the field of genetics and genomics. These advances have had a positive impact on molecular breeding (Mukhopadhyay et al., 2020). Technological advancements that enable the cost-effective collection and use of genomic information, including data on the microbiome, transcriptome, genomic, and epigenomic levels of complexity, will play a significant role (Georges et al., 2019).

Mendel's nineteenth-century discoveries regarding the inheritance of characters in plants are unquestionably the foundation of classical genetic knowledge. Concepts related to genes and chromosome theory solidified in the early 1900s. Subsequently, discoveries regarding gene regulation, the unraveling of the genetic code, and advancements in the chemical basis of inheritance during the 20th century signaled the advent of genomics and the creation of genomic tools in animal science. The whole human genome was sequenced in the early 21st century, and the ultimate use of this knowledge—the development of gene-editing tools—was a significant advancement in genomics. This resulted in the 2020 Nobel Prize in Physics being given to the female researchers Charpentier and Doudna (Bateson, 1902; Bovine Genome Sequencing and Analysis Consortium et al., 2009; Fleischmann et al., 1995; Gayon, 2016; Gayon and Burian, 2000; Gutierrez-Reinoso et al., 2021; Hu et al., 2020; International Human Genome Sequencing Consortium, 2004; Jacob and Monod, 1961; Johannsen, 1926; Lenay, 2000; Maltecca et al., 2020; Matukumalli et al., 2009; Meuwissen et al., 2001; Moore and Hasler, 2017; Ricroch, 2019; Sanger and Thompson, 1952; Schrödinger, 1956; Shendure et al., 2017; Watson and Crick, 1953). In the 1980s, the field of genomics emerged as a promising new discipline that held the promise of identifying the genes and mutations responsible for the domestic biodiversity. This would provide insight into the molecular architecture of the traits subject to artificial selection and enable the development of more efficient marker assisted selection. After 25 years, the repertoire of genomic techniques has significantly expanded, encompassing next-generation sequencing and high-throughput SNP genotyping. Due to the near-triviality of identifying the underlying genes and mutations causing Mendelian traits, including monogenic defects, recessive defects can now be managed with unprecedented efficacy. Breeding cattle is being revolutionized by genomic selection, beginning with dairy cattle, thanks to the availability of medium- and high-density SNP arrays and sophisticated statistical techniques. As it becomes possible to imputation of genome-wide SNP information from resequencing larger cohorts, methods for the identification of quantitative trait nucleotides (QTN) will become more effective (Womack, 2012). Genomic technologies were slow to emerge at first, but in the past ten years, they have advanced significantly. Genomics uses high throughput molecular data to examine the structure and function of entire genomes, in contrast to genetics, which is generally defined as the study of inheritance using traditional theoretical concepts and models. At first, a variety of molecular markers were employed for testing genetic defects and confirming parentage. The SNP is a particular kind of genetic marker that can be found in DNA sequences where distinct nucleotide bases occur at specific positions. Since SNP markers are frequently either directly linked to or linked to a large number of the genes responsible for phenotypic variation, they currently provide the information needed for genomic selection. They are also inexpensive, highly prevalent in the bovine genome, stably inherited, and suitable for high throughput analysis (Baes et al., 2018).

Cattle breeding now heavily relies on genomics, or genetic analyses using markers dispersed throughout the entire genome. More than 8.5 million animals had been genotyped as of December 2023, according to the US dairy cattle evaluation conducted by the Council on Dairy Cattle Breeding (Council on Dairy Cattle Breeding, 2024). The purpose of this data collection is genomic selection, which is the assessment of animals through genome-wide DNA testing, and was introduced in the United States in 2007 [reviewed by Wiggans et al. (2017)]. By estimating a breeding value, or a prediction of the trait values of an animal's offspring, based on measurements on the animal itself and its relatives, genomic selection expands on the practice of genetic evaluation. Genomic selection enriches the assessment with molecular data through genome-wide DNA markers (Johnsson, 2023). This manuscript aimed to review recent advances in genomic technologies for cattle breeding and future prospects in this field.

## 2 Cattle genome sequencing

### 2.1 Advancements in bovine genome assembly techniques and resources

Leveraging the many resources at its disposal, the massive project of sequencing and assembling the bovine genome served as the foundation for future research. The rhesus macaque genome's genome was assembled using a combination of the whole genome shotgun (WGS) local assembly, which is utilized for many other animal genomes, and the bacterial artificial chromosome (BAC) plus WGS local assembly, which is used for rat and sea urchin genomes (Womack, 2012). Recent updates to the genome annotation have been made possible by the extensive and cooperative genome research projects being conducted in Australia, the United States, and Europe. There are currently several databases on the genomes of cattle. Databases of cattle variation, viz. Variation Ensembl (https://www. genome variation map (http://bigd.big.ac.cn/gvm/), NCBI dbSNP (https://www.ncbi.nlm.nih.gov/snp), ensembl.org/info/genome/variation/sources_documentation.html? redirect5no#bos_taurus), etc. include important details about the genes and their variations, orthologs of human disease genes, SNPs and QTLs, etc. The collaborative Bovine Genome Project, with funding from the United States, Canada, the United Kingdom, France, Australia, and New Zealand, has sequenced, analyzed, and partially annotated the taurine genome (Burt, 2009; Mukhopadhyay et al., 2020). The average length of a mammalian genome is approximately 100 million base pairs (100 Mb), with sequences typically consisting of three billion base pairs (three gigabases, or Gb) in total. Read lengths of sequences ranging from 25 to 1000 base pairs are produced by sequencing technologies. The process of merging numerous short sequences (reads) into a single, lengthy consensus sequence is known as genome assembly. This procedure is invariably a compromise, as assembly techniques are chosen to ensure uniform application across the entire genome. Aggressive merging of the sequences may result in false joins under certain circumstances. Alternatively, the sequences can be cautiously combined, which would result in fewer false joins but leave some sequences unjoined (Womack, 2012). Among the most popular approaches for assembling genomes are the combined, WGS, and hierarchical methods. The human genome was sequenced using the hierarchical method, which involves isolating, mapping to the genome, and sequencing each BAC individually (Lander et al., 2001). The hierarchical method has the advantage that each BAC contains DNA from a single haplotype, and for a given level of sequence coverage, the assembly within a BAC is more contiguous and accurate and prevents conflicts caused by polymorphisms. By eliminating the need to build libraries and clone BACs, the whole genome shotgun or WGS method lowers the cost of genome sequencing while also preventing biased representation in sequences that do not clone in BACs. These benefits come at a price, though, as the WGS method struggles more to handle genomic features like polymorphisms and repeats, which make real genomic sequence assembly more challenging than random sequence assembly (Womack, 2012). The Baylor College of Medicine Human Genome Sequencing Center assembled the bovine genome. The sequence assembly strategy, which combined techniques from the hierarchical BAC clone and WGS approaches, was implemented (Liu et al., 2009) that were comparable to the methods employed to put together the genomes of sea urchins (Sea Urchin Genome Sequencing Consortium et al., 2006) and rats (Gibbs et al., 2004). Many of the BACs for the bovine project were sequenced in groups or pools rather than individually in order to save costs, similar to what happened with the sea urchin (Sea Urchin Genome Sequencing Consortium et al., 2006). As the project progressed, multiple assembly versions were created using various data and methodologies and employed for various analyzes. The assemblies are listed in Table 1 along with a brief description of each.

**Table 1.** A list with descriptions of the assemblies of the bovine genome.

Assembly version	Public release year	Description
Btau_1.0	2004	Initial access was granted by a low coverage WGS-only assembly
Btau_2.0	2005	Early access was granted to WGS-only assemblies
Btau_3.1	2006	Most genome analyses employ a combined BAC and WGS assembly
Btau_4.0	2007	additional sequence that was positioned using a different map
Btau_4.2	2009	The corresponding draft sequences from Btau_4.0 were substituted with finished BACs
Btau_4.5	2009	Integrated SOLiD data for scaffolds, added contigs
UMD3.1	2010	Enhancing assembly continuity through the application of a graph-based assembly method
Btau_4.6.1	2012	Using contigs from Btau2.0 to fill the gap in Btau4.0/3.1
Btau_5.0.1	2015	19x PacBio data with UMD 3.1 using PB Jelly is the most improved version that has been utilized
ARS-UCD1.2	2018	Employing techniques for long-read sequence assembly

WGS: Whole genome shotgun; BAC: Bacterial artificial chromosome. Adapted from Womack (2012) and HGSG Bovine Genome Project page (https://www.hgsc.bcm.edu/other-mammals/bovine-genome-project).

### 2.2 Revolutionizing bovine genomics through the 1000 bull genomes project

The 1000 Bull Genomes Project is a groundbreaking initiative that aimed to sequence the genomes of 1000 bulls from different cattle breeds worldwide. This project has played a critical role in the development of bovine SNP chip arrays due to the wealth of genetic data it provided. By sequencing the genomes of 1000 bulls from different cattle breeds, the project has provided a comprehensive understanding of the genetic diversity in cattle populations. This genetic diversity is crucial for developing robust SNP arrays that can capture the breadth of genetic variation within and between breeds. The project has facilitated the discovery of a large number of genetic markers, including SNPs, which are highly informative for studying traits of interest in cattle. These markers serve as building blocks for designing SNP chip arrays that can be used for genomic studies, breeding programs, and other applications. The large amount of genomic data generated by the project has allowed researchers to identify and select the most informative and breed-specific SNPs for inclusion in SNP chip arrays. This selective process ensures that the SNP arrays provide accurate and relevant genetic information for diverse cattle populations. Bovine SNP chip arrays derived from data from the 1000 Bull Genomes Project have revolutionized genomic prediction and selection strategies in cattle breeding. These arrays enable the precise identification of genetic markers associated with economically important traits, enabling breeders to make informed decisions to improve livestock productivity and health. The data from 1000 Bull Genomes Project has greatly sped up the process of identifying harmful mutations that cause hereditary illnesses and embryonic lethals. The availability of bovine SNP chip arrays developed as part of the 1000 Bull Genomes Project has accelerated research in various areas such as genomics, animal breeding, and animal genetics. The project has been used to improve the accuracy of within- and across-breed genomic selection with sequence variants, particularly for breeds not in the reference population. Researchers can now conduct in-depth studies of the genetic basis of complex traits, population structure, and evolutionary history of bovine species (Hayes and Daetwyler, 2019).

### 2.3 Impacts and challenges of next generation sequencing (NGS) in genomics

After the Sanger and the Maxam and Gilbert chemical degradation sequencing method, massively parallel sequencing technology was developed. This technology is known as NGS. NGS has now become a widely used tool in many biological science fields because of its low cost. Genomics data generated by NGS can be utilized to identify genetic variants associated with changes in function. The organization, structure, function, and evolution of livestock animal genomes have all been better understood thanks to NGS. Among its many advantages over other techniques is the high resolution view of the DNA/RNA sequence that NGS offers. Understanding the genetic underpinnings of an animal's behavior and interactions with its surroundings can be started with NGS. It is currently widely used to investigate intricate features in various species. It is anticipated that NGS will lower the total cost of cattle production, boost yield, enhance milk and meat quality, improve disease resistance, and enhance cattle reproductive health (Sharma et al., 2017). The study of rare variants is made possible by NGS technology. NGS technology also makes it possible to search for uncommon variants and identify a wide variety of variants, including SNP and structural variation (Chen, 2011). Finding variations for rare diseases with Mendelian inheritance may be the largest achievement of NGS (Londin et al., 2013). Our capacity to identify variants linked to traits and diseases is greatly impacted by NGS technology (Edwards et al., 2014). New avenues for the identification of DNA building have been made possible by advancements in the implementation and creation of whole genome sequencing (Feuk et al., 2006). NGS is quickly rising to the top of the genetic diagnostics toolkit. But worries have been expressed regarding the volume and complexity of data for the whole genome sequence, which could make the method of interpreting the relationship between genetic variants and diseases ineffective (Goldstein et al., 2013). Millions of genetic variations can be produced by NGS technology and are widely distributed throughout the genome (Luo et al., 2012). As a result, this sequencing technique produced a lot of data. Nevertheless, the entire potential of the genome and epigenome data from NGS cannot be fully utilized by the computational techniques available today. New and improved tools and systems are therefore required (Chaitankar et al., 2016). Bioinformatics techniques for data analysis and storage represent the primary obstacle to NGS (Blaby-Haas and de Crécy-Lagard, 2011; DePristo et al., 2011; Khanzadeh et al., 2020; Londin et al., 2013). Conversely, significant obstacles to the NGS data association test include rare variants with high volume, sequencing errors, and missing data. The Type I error rate and test power for phenotype-genotype correlation are significantly impacted by these difficulties (Khanzadeh et al., 2020; Luo et al., 2011).

Even though NGS technology revolutionized sequencing by providing unprecedented depth and accuracy along with a massive sequencing capacity, it still exhibits significant limitations, the primary one being the production of short reads. All NGS platforms use a technique known as "short-read sequencing," which necessitates the use of complex post-processing pipelines and specialized bioinformatics tools. This increases the average analysis time and makes manipulating high-throughput data more difficult.

### 2.4 Revolutionizing genomic sequencing with third-generation sequencing technologies

Third-generation sequencing emerged in the early 2010s with the introduction of a new set of sequencing methodologies offered by two platforms: Oxford Nanopore Sequencing (ONT) and Pacific Biosciences (PacBio). Third-generation sequencing, a cutting-edge technique that ushered in a new era in sequencing, overcame the limitations of NGS technologies. Third-generation sequencing results in long reads with an average length of more than 10 kb, which is different from NGS platforms that produce relatively short reads (up to ∼600 nt). This is due to improved sequencing chemistry (Goodwin et al., 2016; Michael et al., 2018). Given that it significantly improved the quality of genome assembly and genome structure analysis, the considerable lengthening of the generated sequencing reads is thought to be the most beneficial aspect of third-generation sequencing technologies (Michael et al., 2018; Roberts et al., 2013). More specifically, longer read lengths generate more contiguous genome reconstructions because they function as more representative chromosomal elements (Michael et al., 2018). Long reads made it easier to characterize large insertions, deletions, translocations, and other structural changes that might occur throughout genomes, according to studies on variation analysis (Athanasopoulou et al., 2021). The Bovine Long Read Consortium (BovineLRC) concentrates on characterizing the structural variation of the bovine genome by sequencing cattle at the population scale using long read sequencing technologies (Nguyen et al., 2023). Long-read assemblies from the BovineLRC will make it possible to annotate the genome more precisely, allowing for the identification of genes, regulatory elements, and structural variants. The 1000 Bull Genomes Project's identification of genetic variation will require the interpretation of this improved annotation. Real-time and single-molecule sequencing are the two most distinctive aspects of third-generation sequencing technologies. Less bias and more homogenous genome coverage are made possible by third-generation sequencing techniques' lack of polymerase chain reaction (PCR) amplification, in contrast to NGS, where it is a necessary step in the experimental process (Goodwin et al., 2016). Long-read technologies are excellent for studying transcriptomes, with the exception of genomic DNA sequencing. This allows for the discovery of novel full-length transcript variants and gene fusions that are not detectable with NGS techniques (Roberts et al., 2013). Furthermore, because third-generation sequencing platforms make it possible to explore complex transcriptomes more effectively and easily without the need for specialized assembly bioinformatics tools, they have become the de facto "gold-standard" technology for whole-transcriptome sequencing (Athanasopoulou et al., 2021). Importantly, lengthy experimental procedures, big equipment, and intensive bioinformatics analysis are typically associated with short-read methods. It takes longer to complete the experimental process and complicates the post-processing analysis because of these NGS approach characteristics (Quick et al., 2016; Sharon et al., 2013). Third-generation sequencing platforms, on the other hand, have created new sequencing protocols and, as a result, have streamlined the library construction processes, reducing the amount of time needed for preparation and simplifying the sequencing runs process (Athanasopoulou et al., 2021).

With the major sequencing techniques of DNA-seq and RNA-seq, the development of new research areas was made possible by the evolution of third-generation sequencing (Athanasopoulou et al., 2021). Third-generation sequencing platforms can decipher even the most difficult regions of complex eukaryotic genomes (Jain et al., 2015), uncover structural variants that were undetectable by earlier sequencing chemistries (Audano et al., 2019), and perform telomere-to-telomere assemblies of entire chromosomes (Miga et al., 2020), according to a number of studies that have already been conducted. Revolutionary advances in genomic research and expansion of the scope of DNA studies have been brought about by de novo assembly through whole-genome sequencing approaches. To put it succinctly, the use of DNA-seq application makes it possible to characterize previously undefinable genomes and alter current reference genomes. Additionally, full repetitive regions can be distinguished from pseudogenes, haplotypes and alleles can be determined, pathogen detection, epigenetic modification identification, mutational analysis, and a significant number of variations can all be identified using native DNA sequencing (Kono et al., 2019; Rhodas and Au, 2015).

#### 2.4.1 HeliScope

The first single-molecule sequencing attempt was made in 2009 with the release of HeliScope by Helicos Bioscience. This genetic analysis system performed single-molecule sequencing by taking advantage of the fluorescence phenomenon (Pushkarev et al., 2009), and it required a workflow for library preparation to be completed before sequencing. Due to the avoidance of PCR amplification steps, this method produced short reads (∼32 bp), which was a time-consuming process. However, it also had serious limitations regarding high error rates, high cost, and extensive sequencing (Athanasopoulou et al., 2021).

#### 2.4.2 PacBio

The first single-molecule real-time (SMRT) sequencing technology was introduced by PacBio in early 2011 with the release of their PacBio RS sequencer (Zheng et al., 2016). Despite the fact that the first sequencer produced relatively short average read lengths (∼1.5 kb) with high error rates (∼13 %) (Quail et al., 2012), technology improved over time, resulting in the release of a new sequencer called the Sequel System, which became the standard sequencing apparatus of PacBio for genome analysis very quickly. PacBio's Sequel System continued to be improved over time, giving rise to two more Sequel platforms: the Sequel II System and the Sequel IIe System, the latter of which is the most potent platform in the Sequel family (Kingan et al., 2019).

#### 2.4.3 Nanopore

The development of an additional approach, based on a completely different method from Sequel technology, has enhanced third-generation sequencing technologies beyond PacBio's methodology. More specifically, ONT introduced nanopore sequencing in 2014 (Jain et al., 2015), a technique based on the concept first put forth at the end of the 1980s of using nanopores in a membrane to sequence single-stranded DNA or RNA molecules (Deamer et al., 2016). As a consequence of the notable throughput gains made possible by the quick development of ONT chemistries, Nanopore sequencing has become widely used in various research domains.

Advanced research on transcriptomes and particular RNA classes, like mRNAs, has already been made possible by the technological shift from sequencing amplified or non-amplified cDNA molecules to direct RNA sequencing through nanopores. In addition to being an effective technique for studying alternative splicing, finding novel mRNAs [88], and deciphering patterns of gene expression (Athanasopoulou et al., 2021; Depledge et al., 2019; Stark et al., 2019), direct sequencing of RNA molecules—which was initially made possible by third-generation sequencing—allows for the precise identification of RNA modified bases (Price et al., 2020). Numerous classes of RNAs, such as tRNAs (Suzuki, 2021), rRNAs (Sloan et al., 2017), and mRNAs (Gilbert et al., 2016), undergo chemical base modifications. These modifications have been identified as important regulators of pre-mRNA splicing (Adhikari et al., 2016), nuclear export, mRNA stability and localization, and translation efficiency (Roundtree et al., 2017). The most promising technique for determining not only the epitranscriptomic profile but also its various roles on cellular homeostasis and pathophysiology is unquestionably the transcriptome-wide analysis of RNA modifications through ONT's direct RNA sequencing approach. This difficulty stems primarily from the lack of specialized bioinformatics tools (Athanasopoulou et al., 2021). Small RNAs, such as miRNAs and tRNAs, can also be detected using methods provided by nanopore sequencing platforms. This enables the precise characterization of these RNA molecules as well as the analysis of their expression patterns in any sample of interest (Gu et al., 2012; Zhang et al., 2020). By differentiating between miRNA isoforms, identifying epigenetic modifications, and measuring their abundance in cells, third-generation sequencing has improved research on small RNAs (Zhang et al., 2020). Without a doubt, the first step in understanding the regulatory function of small RNAs in cells and determining how they are related to diseases is direct small-RNA sequencing (Zhang et al., 2020).

Many facets of the complexity of the transcriptomes of humans, plants, and animals have been effectively dissected by the development of ONT real-time direct RNA-sequencing techniques. Since it cannot be obtained through any NGS platform, this extremely creative method directly sequences poly *A*+ RNA molecules that flow through the nanopores. As a result, it has become a standout technique for the study of mRNAs. Even with its novel qualities, the direct RNA method needs a large starting dose of poly *A*+ RNA, and it yields much less than the PCR-cDNA and direct cDNA approaches. An effective method for finding full-length mRNAs and discovering new transcripts that can shed light on the characteristics and patterns of alternative splicing is direct RNA sequencing (Need et al., 2012; Stark et al., 2019). Accurate isoform quantification, polyadenylation site characterization, promoter and splice site identification, and—above all—the identification of RNA modified bases are all made possible by it (Garalde et al., 2018; Hussain, 2018; Lorenz et al., 2020; Oikonomopoulos et al., 2020; Soneson et al., 2019; Zhao, 2019).

#### 2.4.4 Exome sequencing

Exome sequencing, as opposed to whole-genome sequencing, focuses on the protein-coding regions, which make up less than 2 % of the entire genomic DNA. Because more samples can be sequenced in a single run at a lower cost with whole-exome sequencing than with whole-genome sequencing, the latter method is characterized by lower depth and coverage. Whole-exome sequencing is a method that has gained significant importance in various research domains. It can be utilized for both clinical and diagnostic objectives and enables the identification of altered bases in coding sequence regions, the detection of copy number variations (CNVs), and the identification of functional variants associated with disease (Need et al., 2012). Third-generation sequencing platforms' technologies have transformed sequencing not only at the transcriptome and genome level but also in the area of targeted sequencing, making it easier to precisely identify particular transcriptomic or genomic regions of interest. Using the CRISPR-Cas9 system and a total of 5000 ng of non-amplified genomic DNA, PacBio technology provides an effective method for sequencing specific genomic regions under that framework (Athanasopoulou et al., 2021).

#### 2.4.5 cDNA sequencing

In terms of cDNA sequencing, third-generation sequencing has become the "gold-standard" method. Third-generation sequencing platforms generate long reads that can detect fusion transcripts or even full-length mRNAs without requiring assembly. Notably, PacBio uses the cutting-edge Iso-Seq Express Template Preparation method to prepare and sequence cDNA libraries, which makes it possible to sequence RNA molecules indirectly. Conversely, transcriptome profiling and alternative splicing event detection can also be accomplished with nanopore cDNA sequencing. The fact that both the Illumina and Ion Torrent platforms require a significant amount of RNA as starting material makes this one of the most basic benefits of third-generation sequencing over NGS. In the case of difficult samples (e.g., wastewater samples) could be the most detrimental restriction. In addition to this strategy, ONT's direct cDNA sequencing method is a dependable option for cDNA molecule sequencing devoid of amplification (PCR-free), albeit it necessitates a larger amount of input. When analyzing the mRNAs in a sample of interest for differential expression, direct cDNA sequencing is the best approach. However, it is not very effective in identifying novel mRNA transcripts that are scarcer (Athanasopoulou et al., 2021).

#### 2.4.6 Graph genomes

Graph genomes is an emerging field that seeks to include polymorphic haplotypes and genetic variation as alternate pathways in a single genome graph representation. This has the benefit of improving read alignment accuracy even for reads that do not exactly match a linear reference; instead, they may perfectly match a path through the graph. Utilizing such genome graphs can improve read mapping and variant calling precision, decrease mapping biases (Biederstedt et al., 2018; Garrison et al., 2018), find ChIP-seq peaks that are not found with linear genomes (Groza et al., 2020; Grytten et al., 2019), and more accurately characterize transcription factor motifs (Tognon et al., 2021), according to a number of recent studies. But as of right now, there aren't many excellent graph genomes accessible. Graph-based techniques make it easier to compare sequencing reads to a genome graph that takes variation into account. This genome graph includes a set of distinct DNA sequences that are not redundant within a species (Crysnanto et al., 2019). Until now, research using graph genomes in cattle has only been able to incorporate variants from short-read sequencing data into the Hereford reference (Crysnanto and Pausch, 2020; Crysnanto et al., 2019) or analyze differences larger than 100 bp between non-Hereford assemblies and the ARS-UCD1.2 reference genome (Crysnanto et al., 2021). These studies showed that, when using the current standard variant calling algorithms GATK HaplotypeCaller19 and FreeBayes20, the variant calls obtained using the graph genome were more consistent between sire-son pairs than those obtained using the linear Hereford reference, despite the fact that they were not able to capture wider diversity in cattle. Thus, graph genomes may facilitate the identification of genetic variants, including those that may be responsible for significant phenotypic variations among populations and breeds. However, representative reference sequences are a prerequisite for building high-quality graph genomes, and these have been hard to come by for cattle that are not European. European breeds, comprising only 8 % of the global cattle population, have a dominant presence in current genetic resources. This has had a negative impact on research concerning other significant cattle breeds worldwide, particularly African breeds which have limited genomic resources despite their crucial role in the economies of the continent. In order to address this problem, Talenti et al. (2022) have successfully generated assemblies of African breeds and integrated them with genomic data for 294 diverse cattle. This comprehensive graph genome now incorporates global cattle diversity and presents an enhanced and more inclusive reference assembly, thereby advancing global cattle research efforts. Furthermore, Crysnanto et al. (2021) constructed a pangenome from six reference-quality assemblies derived from yak and taurine and indicine cattle using a bovine multiassembly graph. Compared to the reference genome of *Bos taurus*, the pangenome exhibits an extra 70,329,827 bases. Through their multiassembly approach, researchers discovered that yak and indicine cattle possess 30 and 10.1 million bases respectively, that are exclusive to their genomes. Additionally, each taurine assembly reveals between 3.3 and 4.4 million bases that are unique to them. Additionally, their findings open up a previously untapped source of variation for genetic studies and offer strategies and a framework for creating and utilizing a more varied reference genome. Three approaches to creating pangenome graphs from multiple genome assemblies were contrasted by Crysnanto et al. (2022). The pangenome is constructed by Minigraph by performing an approximate mapping of assemblies to a backbone genome. Cactus and pggb, on the other hand, use reference-free base level alignment to create pangenome graphs. According to their findings, compared to the minigraph pangenome, pangenome graphs created through base-level alignment have 40 % more small variations. A nearly identical set of structural variations are revealed by Cactus, pggb, and minigraph. Graphs created using base-level alignment typically have more precise breakpoints and allelic paths than those inferred from minigraph's approximate mapping (Crysnanto et al., 2022).

## 3 Cattle SNP arrays

One of the initial objectives for applying the bovine reference genome to the cattle industry as a whole was parentage verification. Blood typing was the first method of verification and was effective in identifying discrepancies in the expected paternal lineage. Prior to the discovery that short tandem repeat loci, or microsatellites, are more precise and efficient, this technique was the gold standard for parental verification for more than 50 years (Fries et al., 1990; Glowatzki-Mullis et al., 1995). SNPs were discovered by comparing the reference genome's genome sequence to those of other animals, and by the mid-2000s, innovative cattle producers were using them (Heaton et al., 2002). A type of DNA microarray called an SNP array is used to identify polymorphisms at particular SNP loci within or between populations. Essentially, probes (i.e., DNA fragments of known sequence) impregnated on silicon chip to allow for hybridization hydrogen bonds between the complementary cDNA/cRNA (target) sequence that has been fluorophore-prelabelled. An automated scanner is used to determine the amount of fluorescence, or signal, that each spot (or feature) emits. The degree to which a number of molecules have hybridized determines the fluorescence intensity (Mukhopadhyay et al., 2020). At first, it was predicted that each SNP genotype would cost $1, making SNP genotype technology far more expensive than microsatellite technology as it is today. Scientists at the USDA Meat Animal Research Center (MARC) released a first panel of 26 extremely informative SNPs to be used as parentage markers. The MARC bovine expressed sequence tag (EST)/SNP linkage mapping project and ongoing haplotype studies of seven genes were the sources of these SNPs (Heaton et al., 2002). SNP parental verification gained traction in the field more slowly than the more widely used microsatellite-based analyses because of the previously mentioned high costs, challenges in implementing new technology, and reluctance to abandon established data sets and methodology (Vignal et al., 2002). Imputation from SNP to microsatellites was introduced, which was a helpful step in getting the industry to move away from using microsatellites. This tool made the switch to the new technology easier because it did not need genotyping sires who had previously undergone microsatellite testing (McClure et al., 2012). SNP have significant advantages over microsatellites, despite their initial higher costs. These advantages include reduced mutation rates, more efficient laboratory procedures, and more sophisticated data interpretation (Krawczak, 1999; Kruglyak, 1997). Furthermore, compared to microsatellites, which had some legacy data inconsistencies in parental lineage identification, they are easier to standardize (Fries and Durstewitz, 2001). By multiplexing thousands of SNP sites on a single assay, oligonucleotide arrays for DNA analysis, which were developed in the 1990s, allowed for higher-throughput sample processing (Pease et al., 1994). The development of genotyping assays in the cattle industry would be greatly aided by this technology. SNP genotyping became less than half as expensive as microsatellite genotyping by 2009. This was due to significant cost reductions (Tokarska et al., 2009). A thorough analysis contrasting the performance of SNP panels and microsatellites has already been released (Fernández et al., 2013). The information required to create more comprehensive SNP resources was made available by the first releases of the cattle draft genome and the underlying sequence data. Approximately 10,000 SNP marker sites made up the Affymetrix 10 K cattle genotyping chip (Affy 10 K), which was created by Affymetrix to provide initial parentage verification (Table 2). The Affy 10 K was developed at the same time as the Btau2.0 reference assembly. It consisted of SNP markers spaced approximately 1.71 SNP/cM on average (Daetwyler et al., 2008). Eight percent of the markers on this chip, which were provided by the Commonwealth Scientific and Industrial Research Organization (CSIRO), were found to be within genes. Ninety-two percent of the markers were identified from the WGS sequence data of four cattle breeds: Holstein, Angus, Limousin, and Hereford (albeit perhaps not encoding genes; see http://tools.thermofisher.com/content/sfs/brochures/bovine10ksnpdatasheet.pdf). Since it was challenging to assign marker SNP to physical cattle chromosomes in the draft state of the Btau2.0 reference assembly, about 5000 of the marker SNP were not assigned to chromosome linkage groups, severely limiting the chip's usefulness. There were inconsistent results in the initial trials that used this assay to find QTLs (Hayes et al., 2006), probably because there are more markers spaced apart and fewer polymorphic sites from different cattle breeds are represented. Notwithstanding this obstacle, further attempts to create improved genotyping assays would ultimately be successful in producing the Btau3.1 and 4.0 assemblies. New breed-representative polymorphic SNP sites that were uniformly distributed across the cattle genome were sought after. The use of high-throughput genotyping in the cattle industry would be aided by the publication of new techniques, data sets, and commercial products by a number of cattle genomics consortia and participating geneticists in the two-year period between 2008 and 2009. Twenty-six WGS sequence reads for 24 different breeds and two subspecies of cattle were included in a data set made available by the Bovine HapMap Consortium (Bovine HapMap Consortium et al., 2009). The representation of genetic variant sites from the Bos taurus indicus subspecies and previously unsequenced breeds was enhanced by this data set. A new technique for reduced representation library sequencing that is effective for sequencing pooled samples (Van Tassell et al., 2008) made it possible to quickly identify SNP for assay design. The breadth of population variant coverage was increased by more thoroughly sequencing more individuals at a fixed cost by concentrating on a subset of the genome. Lastly, a technique for designing genotyping chips by choosing uniformly spaced polymorphic marker sites (Matukumalli et al., 2009) to create the BovineSNP50, a new SNP chip for cattle. The ag-genomics industry, worth millions of dollars, was created when the products of these innovations were quickly embraced not only by the cattle research community and industry, but also by nearly all agricultural species communities. Concerns about the BovineSNP50′s marker density cast a shadow over the project's success. The research community was divided on whether breeding value estimations could be significantly improved by a higher-density assay (>100,000 genetic markers) (Meuwissen and Goddard, 2010) or if there would be no benefit at all (VanRaden et al., 2011) among cattle breeds that are dairy. The creation of the BovineHD (also known as HD) genotyping array in 2011 would supply the information needed to respond to this query (Matukumalli et al., 2011). Higher-density genotypes did not appear to add much value in subsequent analysis of HD genotypes from Holstein cattle (VanRaden et al., 2013); nevertheless, improved genetic assessment of these breeds was made possible by greater representation of polymorphic markers from indigeneous cattle breeds (Nayee et al., 2018). Developing lower-density SNP arrays that could be genotyped at a lower cost to the industry and be useful for genomic selection also sparked a lot of interest. In order to produce low-density chips that were cost-effective for population-scale genotyping, quantitative techniques defined a smaller SNP set for particular breeds (Wiggans et al., 2012). Enhancements in the process of imputed these markers to arrays with a higher density (VanRaden et al., 2013) encouraged cow and calf genotyping and made these lower-density chips usable for regular genetic assessments. The Council on Dairy Cattle Breeding states that as of September 2019, 3,541,090 SNP genotypes have been gathered from dairy cattle alone (https://queries.uscdcb.com/Genotype/cur freq.html).

**Table 2.** A list of commercially available bovine SNPs that are used for genotyping assays in genomic selection.

Genotyping chip^1^	Marker count	Assembly	Contents
Affymetrix GeneChip bovine mapping 10 K	9919	Btau1.0	92 % of four cattle breeds were subjected to Baylor whole-genome shotgun (WGS) sequencing. 8 % of markers specific to CSIRO genes.
Affymetrix GeneChip bovine mapping 25 K	25,000	UMD3.1.1	10,000 unique SNPs from the sequencing project were included in this GeneChip in addition to an extra 15,000 novel SNPs.
Affymetrix axiom BOS 1 array plate	640,000	–	Three distinct next-generation sequencing platforms were used in the design of the Affymetrix BOS 1 assay to sequence individuals from fifteen different breeds of B. *taurus* and B. *taurus indicus* cattle. Using sequenced animals and animals from the HapMap project, a prescreening array was developed with the aim of validating and estimating MAF for nearly four M SNPs.
Illumina SNP50 BeadChip	54,001	Btau3.1/4.0	50 % is decreased representation library SNP. 25 % is the Bovine Hapmap. Four cattle breeds' Baylor WGS accounts for 20 % of this.
Illumina BovineHD BeadChip	777,962	UMD3.1	Seven percent are Illumina SNP50 BeadChips; ninety-three percent have 180 × coverage WGS of 20 cattle breeds.

Adapted from Womack (2012) and Bickhart et al. (2020).

## 4 Genomic selection and genome-wide association studies (GWAS) in cattle

### 4.1 Revolutionizing cattle breeding with genomic selection

The process of breeding improved genetic materials in animal breeding programs has been revolutionized by the process of genomic selection. By lowering the generation interval and bull proofing costs, genomic selection has increased animal productivity. Utilizing marker data to estimate breeding value in the absence of gene location information is the fundamental tenet of genomic selection. Compiling the phenotypic and genotypic data of the reference population is the first stage of genomic selection. All animals within the reference population have their entire genome's SNPs genotyped in order to provide genotypic information (Boichard et al., 2016). Even though large-scale population genotyping is costly, results will be more accurate if there are more animals in the reference population (Li, Bao, & Sun, 2011). Next, the gathered phenotypic and genotypic information is utilized in order to derive a predictive equation for calculating genomic estimated breeding value (GEBV) (Fernandes Júnior et al., 2016). Candidates for selection who have marker genotype data but unknown phenotypes are then subjected to these effects. When compared to traditional selections, genomic selection has a high level of efficiency for sex-limited (milk yield), low heritable, or poor predicted breeding value traits such as fertility traits (Hiendleder et al., 2005). Prior to the advent of genomic technology, thousands of bulls were used for artificial insemination and progeny testing, as well as a vast phonotypic record, in order to improve genetic diversity. However, progeny testing is no longer required with the development of genomic selection, which simplifies and lowers the cost of the selection process; the annual genetic trend may double as a result of a significant reduction in generation interval; a greater number of bulls may be selected and marketed as a result of their lower production costs, improving the management of genetic resources and reducing inbreeding trends (Ibtisham et al., 2017). Thus, the most efficient modern breeding technique for producing and choosing superior animals is whole genome selection. Because beef cattle have a longer generation interval than dairy, the beef cattle industry could primarily benefit more from genomic selection than the dairy industry. Traditional methods for conventional evaluation are hampered by the inability to save phenotype data of a sufficient size and value. As a result, genomic selection has the potential to greatly improve genetic gain by boosting selection reliability at a young age (Jonas and de Koning, 2015). On the other hand, compared to dairy cattle, the genomic selection efficiency of beef cattle remains lower. This could be attributed to various factors such as breed heterogeneity, less sophisticated breeding programs and structures, the prevalence of natural service, cross-breeding in commercial herds, and effective population size (Johnston et al., 2012). Compared to dairy cattle, the genomic prediction reliability in beef cattle has been lower (Van Eenennaam et al., 2014). Because there are fewer and lower-quality beef cattle than dairy cattle, the reliability is lower. Furthermore, compared to dairy cattle, the objective population and validation animals may have a weaker relationship with the reference population in the case of beef cattle. Combining data from different breeds and/or nations may improve prediction accuracy by resolving the issue of small reference populations (de Roos et al., 2009). The effectiveness of genomic selection in beef cattle may be increased with improved genotypic data collection and phenotyping (Ibtisham et al., 2017). Embryo technologies are a tool that can be used, much like genomics, to increase genetic gains by shortening the generation interval and intensifying dam selection so that more calves are born from the best dams. Rather than being additive, genomic selection and embryo technologies work in tandem to make both more effective. Specifically, while genomics helps make embryo technologies more successful, embryo technologies also help make genomics more effective (Miller, 2022). It was stated that the selection of American Angus cattle has significantly improved thanks to genomics, with moderate accuracy available for all measured traits. This allows for the use of younger sires without the requirement for progeny testing, and the recent trend of sire average ages being lower validates this. Since nearly half of all females are genotyped annually and more genetic differences among young females are being discovered, genomics supports the efficient use of embryo technologies by increasing the intensity of selection and reducing inbreeding (Miller, 2022).

### 4.2 Exploring genetic variants with high-throughput genotyping and GWAS

High throughput genotyping technologies have made it possible to find novel genetic variants, like SNPs, linked to economically significant traits in cattle. Because SNPs are widely distributed throughout the genome and are heritable, they are the preferred genetic markers. An increasing number of studies are using GWAS as their standard experimental method to look into SNP markers linked to different economic traits in animal production. Using statistical models, this method links the genotype data to the phenotype in order to look into the genetic variants that cause the desired traits (Mkize et al., 2021). The goal of GWAS method is to determine which detectable common genetic variants (SNPs, insertion-deletions (InDels)) in an individual are significantly associated with the trait under investigation. With the GWAS, animals with different traits have their DNA profiles compared (disease-prone, low productivity, reproduction, or growth parameters in comparison to the control group (individuals in good health or with normal parameters)). Thousands of individuals (split into treatment and control groups) have their DNA samples examined using microarray analysis (or NGS followed by in silico analysis) to find particular SNPs that are more common in a particular group. Thousands should make up the sample size in order to improve the statistical analysis's robustness. It has been reported that the odds ratios for the associations between SNPs and causal variants are typically low, less than 1.5. In order for the test to pass the multiple testing corrections, a larger sample size increases the test's power (Mukhopadhyay et al., 2020). The genetic architecture of complex traits, low allele frequencies, effect size, genetic heterogeneity, low linkage disequilibrium (LD), and missing genotypes present challenges for GWAS (Korte and Farlow, 2013). The GWAS still faces significant technical and analytical difficulties, such as the need to account for genetic variance for complex traits, multiple test corrections, missing loci or blocks, low power to identify sites with low effect, stratification finding risk, overestimation of haplotype effects, poor model fitting, insufficient sample size, low-density SNP coverage, and bringing out rare variants and unknown CNV effects (Clarke and Cooper, 2010; Gibson, 2010; Kadarmideen, 2014; Khanzadeh et al., 2020). Nowadays, GWAS has brought increasing attention to various types of genomic variants, such as haplotype, InDels, CNVs, haplotype, presence/absence variations (PAVs) based on pan-genome, and even epigenomic markers (Tan et al., 2023). For several different breeds, multiple variant profiles have been created (Huang et al., 2021). A total of 14 CNVs, for instance, were discovered to be connected to characteristics related to the health of the cows' hooves, such as heel horn erosion, digital dermatitis, and interdigital dermatitis (Butty et al., 2021). Thankfully, the GWAS data are freely accessible in public databases following their publication. GWAS has made it possible to obtain larger sample sizes with genomic and phenotypic information because of the accumulation of shared data. Many of the cattle-related GWAS data have been entered into the GWAS Atlas database thus far (Liu et al., 2023). Furthermore, additional GWAS research has been made easier and faster thanks to the foundation of a multi-omics database and the Functional Annotation of Animal Genome project database (Tan et al., 2023). The Animal Imputation Database (https://ngdc.cncb.ac.cn/data-basecommons/database/id/6833), which covers 13 species and has 2265 samples, is devoted to gathering publicly available genomic sequencing data obtained from livestock animals in order to build high-quality reference panels (Yang et al., 2020). The IAnimal Database e (https://ianimal. pro/index), for instance, houses the raw data obtained from whole-genome sequencing, RNA-seq, ChIP-seq, and ATAC-seq analyses of 21 animal species (Fu et al., 2023). Furthermore, a greater understanding of the genetic basis of traits through GWAS is being facilitated by the Ruminant Genome Database (http://222.90.83.22:88/ code/index.php/RGD) (Fu et al., 2022), other databases or datasets with genomic variation information (Chen et al., 2020; Hu et al., 2022), transcriptomic characteristics (Goszczynski et al., 2021; Zhang et al., 2022), genome functional annotation (Chen et al., 2022; Halstead et al., 2020; Kern et al., 2021), and pan-genomes (Zhou et al., 2022). However, population stratification—which results from a variety of ancestral kinship—is an unavoidable issue that could lead to false correlations (Ma et al., 2012). The predominant genotypes found in particular populations may mask the actual GWAS hits. In multiple population studies, population stratification is typically blamed for variants or GWAS signals with small effect sizes (McClellan and King, 2010). Improvements in algorithms and software, such as principal component analysis, have enabled better control (van den Berg et al., 2019). Additionally, GWAS hits can be validated through family‐based association studies, such as QTL mapping or LD and linkage analysis approaches, which are useful for controlling population stratification (Ott et al., 2011).

### 4.3 Genomic prediction techniques in GWAS

#### 4.3.1 Linear and non-linear methods

Numerous statistical techniques have been developed to predict the effects of markers and, consequently, the genomic breeding values of individuals. According to Daetwyler et al. (2010), these models can be broadly divided into linear and non-linear techniques. These techniques include Bayesian methods with different prior assumptions and Genomic Best Linear Unbiased Prediction (GBLUP). However, non-additive effects like epistasis and genotype-to-genotype interactions were not taken into account by these traditional methods (Bayer et al., 2021) which can significantly impact animal species' phenotypes. Furthermore, the "curse of dimensionality," or "large P, small N" paradigm, is made worse by genotyping's constant supply of marker datasets (Nayeri et al., 2019). As a result, the large volume of noisy data made it difficult for traditional linear models to identify patterns and explain the intricate relationships that were concealed within (Chafai et al., 2023).

For genomic predictions, GBLUP model is currently employed in a two-step (or multi-step) manner (Misztal, 2016). To compute estimated breeding values (EBVs), this means performing the standard BLUP evaluation. After extracting the pseudo-observations for genotyped individuals, the EBVs are de-regressed (dEBV) and subsequently utilized as input variables for genomic predictions (Misztal et al., 2009, 2013). Nevertheless, the two-step methodology for genomic selection using the genetic relation matrix (G) involves multiple approximations and is complex (Misztal et al., 2013). The approximate accuracy of EBVs and other estimated effects are prerequisites for pseudo-observations. The accuracy of EBVs is decreased by all approximations, which may lead to an increase in GEBVs (Misztal et al., 2009). Additionally, the adoption of the single-step GBLUP methodology (ssGBLUP) has been aided by the fact that only a small percentage of animals in certain developing nations have genotype information available (Misztal et al., 2009). This model predicts the genetic merit of the animal by combining genomic relationships and pedigree information into an H matrix. By utilizing all available data, the model achieves higher accuracy in predicting genetic merit ((Cardoso et al., 2015; Misztal et al., 2009; Valente et al., 2016). It was demonstrated that a ssGBLUP approach can be easy, quick, and precise (Aguilar et al., 2010; Nayeri et al., 2019).

GBLUP represents a linear model, whereas non-linear methods are exemplified by Bayesian approaches (such as Bayes-(A/B/C/etc.)) that employ the Monte Carlo Markov Chain (MCMC) methodology (Gianola et al., 2009; Habier et al., 2011). As Neves et al. (2012) as well as de Los Campos et al. (2013) explain, the primary distinction between these two approaches is their underlying presumptions regarding the impact of SNPs. Similar to the infinitesimal model of quantitative genetics, the GBLUP assumption asserts that the genetic variation for the trait is uniformly distributed across all SNPs on the genotyping panel (Strandén and Garrick, 2009). The GBLUP model is more frequently applied in routine genomic evaluations, where the traditional pedigree-based relationship matrix (A) in the BLUP model is replaced by a genomic relationship matrix (G) among all individuals in the population, which is constructed using high-density SNP genotypes (Ødegård and Meuwissen, 2014, 2015). Although Bayesian methods use the same sampling model, they differ in how priors are adopted (Zhu et al., 2016). For instance, in BayesB, the prior distribution of SNP effects is assumed to be zero with probability of π, normally distributed with a zero mean, and locus specific variance with probability (1−π). In contrast, BayesA assumes that all SNPs have effects and that each SNP has its own variance (Meuwissen et al., 2001). When utilizing MCMC algorithms to implement Bayesian methods for large numbers of SNPs, the process becomes computationally demanding and time-consuming. Thus, a number of iterative (non-MCMC-based Bayesian) techniques, including fastBayesB (Meuwissen et al., 2009), VanRaden's non-linear A/B (VanRaden, 2008), emBayesR (Habier et al., 2007) as well as MixP (Yu and Meuwissen, 2011) were created in order to meet the demands of computing. The previously mentioned techniques yield prediction accuracies that are comparable to those of the MCMC-based techniques and are computationally quick. Non-linear techniques have a greater ability to utilize the LD information obtained from QTL mapping than GBLUP does (Habier et al., 2007). Still, research results show that GBLUP is about as accurate as Bayes-A/B/C methods overall (Hayes et al., 2009; VanRaden et al., 2009). This suggests that there are a lot of QTLs and that, for the majority of traits, the infinitesimal model is roughly accurate (Daetwyler et al., 2010). Consequently, rather than ascertaining the effects of key genes through the use of Bayesian models, an improvement in the accuracy of genomic selection primarily stems from a more precise and improved estimation of the genomic relation matrix (G) among animals (Misztal et al., 2013).

#### 4.3.2 Machine learning (ML) methods

In recent times, there has been much talk in the scientific community about the advancement of machine learning algorithms and the corresponding increase in computational power. ML models are renowned for their extreme adaptability and capacity to uncover patterns in sizable, noisy datasets, like image-based data (Xiao et al., 2015), enormous collections of diverse records (Li et al., 2018), or digital data, which is dramatically growing as a result of developments in computer hardware, computer vision, natural language processing (NLP), and the internet of things (IoT) (David et al., 2019). The development of sequencing technologies has made genomics a field where researchers work with large, complex, redundant, and heterogeneous omics datasets. As a result, numerous studies have looked into the use of machine learning models in genomics (Chafai et al., 2023). The following are some popular machine learning algorithms for genomic prediction: 1. Linear regression, 2. Logistic regression, 3. Decision trees, 4. Ensemble learning (including bagging, random forest, and boosting), 5. Kernel-based algorithms (including reproducing kernel Hilbert spaces (RKHS) and support vector machines (SVM)), 6. Nearest neighbors, 7. Deep neural networks (including multilayer perceptron (MLP), recurrent neural networks (RNN), and convolutional neural networks (CNN) (Chafai et al., 2023). There are numerous significant uses for ML models in the field of genomics. Large datasets of genotypes and phenotypes can be used to train ML using complex algorithms and computational models, which can then be used to predict the breeding values of animals for particular traits. This would facilitate more informed breeding decisions and allow for an accurate selection of animals with the highest genetic merit. In order to predict genomic breeding values, ML models have been effectively applied in dairy cattle (Beskorovajni et al., 2022) and beef cattle (Srivastava et al., 2021). Animals with high genetic potential that exceed the population average can be identified thanks to the estimated GEBVs, which offer an accurate assessment of an animal's genetic potential. As a result, ML models may be very helpful in helping breeders make more accurate breeding choices, which will hasten the genetic advancement (Chafai et al., 2023). Furthermore, to find genetic variants and biological pathways connected to particular phenotypic traits, ML models can be combined with GWAS and population genomics. Zeng et al. (2021) presented a framework for deep learning using the zygosity of SNP data from plants and animals as input to predict quantitative phenotypes of interest and find genomic markers. Additionally, while genotyping large populations can be costly and time-consuming, ML models can be used to impute genotypes with a moderate density. By filling in the blanks, machine learning models are able to generate genotypes with a moderate density. In the beef cattle genomic dataset, this has already been implemented (Sun et al., 2012). When considered collectively, machine learning models seem to be a potent instrument that can facilitate targeted selection, more precise predictions, and a better comprehension of genetic processes. Yet there can be a number of difficulties when using biological data to train machine learning models. The high heterogeneity of the input data, for instance, can be a challenge when combining environmental, phenotypic, and marker data to predict a particular variable. For this reason, pre-processing, which involves scaling, normalizing, cleaning, and formatting the data, is essential. In order to maximize the machine learning model's accuracy and performance, this step makes sure the data is ready. Typically, marker data sets are large and heavily noisy. Overfitting and poor performance can result from using the raw data. Feature selection is therefore essential when working with omics data in order to select pertinent features and remove noise from the model, which in turn reduces the dimensionality of the data. Feature selection can be done in a variety of ways, such as through correlation analysis, statistical techniques, or hypothesis testing. ML models have shown to be incredibly effective at feature selection recently. Filters, wrappers, and embedded methods that combine filter and wrapper methods are the most widely used machine learning-based techniques for feature selection (Tadist et al., 2019). When working with animal species marker data sets, machine learning-based feature selection is frequently employed. To ensure the quality of the data fed to the model, a number of steps should be taken when training machine learning models on biological data. Optimizing the model's performance also primarily involves modifying the hyperparameters and expanding the model's scope using regularization strategies. ML models can be optimized using a variety of methods, including genetic algorithms, random search, grid search, gradient descent, and stochastic gradient descent (Chafai et al., 2023). A summary of recent research on the application of ML methods in genomic studies of cattle is shown in Table 3.

**Table 3.** A summary of recent research on the application of ML methods in genomic studies of cattle.

Study	ML algorithm	Used chip	Breed	No. of animals	Trait(s)/aims
Nishio et al. (2023)	Gaussian kernel, deep kernel, random forest, extreme gradient boost, and convolutional neural network	Illumina BovineSNP50 BeadChip	Japanese Black	10,194	Oleic acid and monounsaturated fatty acid
Lee et al. (2023)	Deep learning networks	Illumina Bovine SNP50 BeadChip	Korean native	10,000	Carcass weight, eye-muscle area, backfat thickness, and marbling score
Ryan et al. (2023)	Random Forest, and partial least square discriminant analysis	Illumina beadchip (IDBV3)	Thirteen breeds	703,078	Determination of breed composition
Shirzadifar et al. (2023)	Eleven ML algorithms	Illumina 50 K SNP BeadChip	Beef cattle	4057	Residual feed intake
Liang et al. (2023)	ST regression and MAK	Illumina 770 K BovineHD BeadChip	Chinese simental	1478	15 slaughter traits
Kasarda et al. (2023)	Random forest	Illumina 50 K SNP BeadChip	17 dairy and beef cattle breeds	1370	Determination of breed composition
Santana et al. (2022)	AdaBoost, Naïve Bayes, Decision Tree, Deep Neural Network, k-Nearest Neighbors, Multi-Layer Perceptron Neural Network, and Support Vector Machine	Illumina 777 K BovineHD BeadChip	Nellore	559	Stayability
Raschia et al. (2022)	XGBoost, LightGBM, and random forest	BovineSNP50 v2 BeadChip	Holstein, Jersey, and their crosses	969 cows and 29 bulls	Milk yield, milk fat and protein contents
Alves et al. (2022)	Random forest	Illumina BovineHD panel	Nellore	3174	Age at first calving
Wilmot et al. (2023)	GRM_SVM and PLS_NSC	The BovineSNP50 Beadchip v2 and 3, the BovineHD Beadchip v12 and the EuroG MD v9-SI and v2	Meuse-Rhine-Yssel, East Belgian Red and White, and Red-Pied of the Ösling	EBRW (*N* = 226), the RPO (*N* = 132), and the MRY (*N* = 292)	To compare the performances of a breed assignment model based on a GRM, combined or not with a machine learning method

### 4.4 Innovative statistical models and algorithms for advanced GWAS analysis

Technology advancements in modeling and computation have made it possible to study multiple data types, covariables, and fixed effects simultaneously, leading to the development of GWAS (Tibbs Cortes et al., 2021). When creating case-control studies, such as those examining health or disease in cattle populations (such as bovine mastitis susceptibility), category data can be analyzed using generalized linear models (e.g., logistic regression), which PLINK software could be used to carry out (Purcell et al., 2007). Various models such as Bayes, FarmCPU, mixed linear model (MLM), compressed mixed linear model (CMLM), general linear model (GLM), and others can be used to analyze continuous variables for non-categorical traits (Liu et al., 2016; Loh et al., 2015; Price et al., 2006; Yu et al., 2006; Zhang et al., 2010). When relatedness influences the traits, which is handled as a random effect in MLM, it is preferred over GLM and exhibits a higher empirical fitting (Yu et al., 2006). To lessen the computational load, numerous statistical models and algorithms have also been incorporated into various programs, including GEMMA, EMMAX, P3D, FaST-LMM, and GRAMMAR-Gamma (Kang et al., 2010; Lippert et al., 2011; Svishcheva et al., 2012; Zhang et al., 2010; Zhou and Stephens, 2012). However, these techniques have the same statistical power as MLM. The majority of earlier research has operated under the assumption that the GWAS model incorporates only the additive effect. But there is also value in the non-additive effect, or epistatic effect (Chimusa et al., 2019). The evaluation of gene-gene or SNP-SNP interactions has proven difficult, primarily because statistical and methodological innovations have not been forthcoming. Nonetheless, this conundrum has been resolved with the development of various software and algorithms, including REMMAX (Wang et al., 2020), MatrixEpistasis (Zhu and Fang, 2018), and Epi‐MEIF (Saha et al., 2022).

Improvements in both the genomic and phenomic domains have been essential to GWAS's growing power. Similar to the exposure variable in Mendelian randomization (MR) analysis, intermediate variables or phenotypes link genetic markers with specialized phenotypes (Tan et al., 2023). Major genes or variants involved in particular traits are difficult to identify due to the low measurement accuracy of common phenotypes in GWAS. Metabolites are thought to be the link between genotypes and phenotypes because they are the byproducts of cellular regulatory processes and represent the ultimate response of biological systems associated with genetic changes (Fiehn, 2002). When compared to common traits, it may be possible to obtain a significant genetic effect from metabolites. This issue might be solved by applying metabolome-based GWAS, which uses hundreds of intermediate metabolites as the phenotypes under study (Suravajhala et al., 2016). Therefore, when enough data are gathered, more GWAS must be carried out in order to connect genetic markers with intermediate phenotypes. The metabolome-based GWAS (mGWAS), proteome-wide association study (PWAS), and transcriptome-wide association study (TWAS) are some of the additional methods. Exploring the molecular mechanisms behind disease resistance and precise nutrition can be accomplished through the potential use of mGWAS. Animal traits may also be influenced by DNA methylation markers and components of the gut flora in addition to conventional genetic markers. The development of the metagenome-wide association study (MWAS) and epigenome-wide association study (EWAS) is a result of the ability to investigate the relationships between animal phenotypes and microbiota features through low-cost, large- or hyper-scale sequencing (Tan et al., 2023). Li et al. (2019) have noted that a key factor in feed efficiency is the microbiota, indicating that a wide range of research examining the regulatory mechanism of focal traits in cattle can make use of the MWAS (Tan et al., 2023). Table 4 presents an overview of current studies on GWAS in cattle.

**Table 4.** A summary of recent research on GWAS in cattle.

Study	Used chip	Country	Breed	No. of animals	Trait(s)
Ma et al. (2023)	Illumina 150 K bovine bead chip	China	Holstein	2029	Milk urea nitrogen
Teng et al. (2023)	Illumina Bovine SNP50Kv1, SNP50Kv2, GeneSeek Genomic Profiler Bovine HD, GeneSeek Genomic Profiler Bovine 100K	China	Holstein	6470	Milk yield, milk fat and protein percentages
Alves et al. (2023)	Illumina BovineSNP50 chip	Canada	Holstein	6849	Fertility and reproduction traits
Won et al. (2023)	Bovine SNP50k chip	Korea	Hanwoo	135	Collagen content traits
Adhikari et al. (2023)	Neogen GGP Bovine 100 K BeadChip	United States	Beef cattle in Hawai'i islands	352	Carcass weight
Jourshari et al. (2023)	Illumina bovine SNP50 chip	Iran	Native cattle of Guilan	72	Abdomen depth, head width, hip width, and withers height
Calderón-Chagoya et al. (2023a)	GGP bovine 150 k chip	Mexico	Simmental and Sambrah	1130	Birth weight, weaning weight, and yearling weight
Li et al. (2023)	80 K SNP panel	United Kingdom	Holstein	2250	Claw horn lesions
Jiménez et al. (2023)	Axiom bovine genotyping v3 array	Spain	Retina	252	Reproductive efficiency
Rocha et al. (2023)	Illumina BovineSNP50 BeadChip v2 (50 K), Illumina BovineHD BeadChip (777 K), GGP Indicus (34 K), Z-Chip (30 K), and GGP Indicus (50 K)	Brasil	Gir	2093	Number of total and viable oocytes, and number of embryos
Haque et al. (2023)	50 K Illumina bovine SNP chip	Korea	Holstein	2329	Body conformation traits
Yu et al. (2023)	600 K high-density chip	China	Qinchuan	254	Body conformation traits
Bejarano et al. (2023)	Illumina BovineSNP50 BeadChip	Colombia	Blanco Orejinegro and Romosinuano	596 and 569	Body weight at different ages
Gao et al. (2023)	Various SNP chips	United States	Holstein	3,649,734	Heifer livability and early calving
Sood et al. (2023)	Illumina BovineSNP50v2 BeadChip	Canada	Canadian beef cattle	1035	Primal cut lean traits
Alam et al. (2023)	Illumina bovine 50 K v.3 SNP chip	Korea	Hanwoo	9302	carcass weight, eye muscle area, backfat thickness, and marbling score
Neustaeter et al. (2023)	50 K and high-density (HD) SNP panel	Canada	Holstein	165	Spastic syndrome
Calderón-Chagoya et al. (2023b)	GGP bovine 150k chip	Mexico	Simmental and Sambrah	547 and 583	Reproductive traits and frame score
Cai et al. (2023)	Illumina BovineSNP50 BeadChip	Denmark	Nordic red	3077	Young stock survival and stillbirth traits
Baba et al. (2023)	Illumina Bovine50 BeadChip or BovineLD BeadChip	Japan	Holstein	1410	Height

### 4.5 Enhancing genomic prediction and accurate imputation in GWAS studies

The findings of the GWAS may improve the precision and robustness of genomic prediction, permit the application of progressively sophisticated models that transcend additive effects, clarify the genetic makeup of specific traits, and ultimately help unravel the biology behind complex traits. To reduce the creation of false-positive and false-negative associations as well as misleading connections to biological processes, the entire process—which includes data generation, quality control, statistical analyses, result interpretation, and connecting findings to biology—should be planned and carried out. False-positive associations have the potential to reduce prediction accuracy if they are prioritized in genomic selection models, or they may cause studies to be misdirected in their search for causal variants, wasting time and resources (Sahana et al., 2023). Allele-calling algorithms and SNP array genotyping technologies available today guarantee accurate marker genotypes for GWAS. Common SNP arrays, on the other hand, only contain a small number of markers and are unable to sufficiently tag millions of sequence variants in cattle genotyping. As a result, the whole-genome sequence needs to be examined in order to identify causal variants. However, only a small number of animals have been sequenced, and this panel's phenotypic makeup makes it inappropriate for GWAS. Consequently, the imputing of whole-genome sequence variant genotypes into the mapping population of animals genotyped with SNP arrays is a growingly popular practice in cattle (Sahana et al., 2023). Using local LD patterns from a reference panel of phased haplotypes, genotype imputation is a statistical procedure to infer missing genotypes in target samples (Treccani et al., 2023). The quantity of significant findings on Mendelian or complex traits was raised by genotype imputation. Not only was genotype imputation used to strengthen GWAS, but it was also used to fine-map variants and validate weak evidence of association, verify or adjust genotyped markers based on their computed probabilities (Marchini and Howie, 2010), and merge several studies into meta-analyses (Zeggini et al., 2008). In order to help manage the growing computational load, a wide range of tools and techniques were quickly developed after the introduction of genotype imputation. Two general categories of imputation techniques are employed in livestock breeding: 1. LD methods based on Mach (Li et al., 2010), Beagle (Browning and Browning, 2013), Impute2 (Howie et al., 2009), and 2. Techniques based on segregation and pedigrees (LE) or a mix of population, segregation, and pedigree data, such as FImpute (Sargolzaei et al., 2008, 2014), PedImpute (Nicolazzi et al., 2013), Findhap (VanRaden et al., 2011), DAGPHASE (Druet and Georges, 2010), and AlphaImpute (Hickey et al., 2012). There are some advantages to population-based imputation techniques. One of the issues is the infrequency of incomplete or nonexistent pedigree information. Comparing these techniques to pedigree imputation yields more accurate imputation for common SNP variants (Cheung et al., 2013). One potential drawback of certain pedigree-based techniques could be their dependence on the presence of dense genotypes for all close ancestors (Hickey et al., 2012). Every imputation technique currently in use is essentially based on looking for comparable haplotypes of the reference panel's observed genotype of animals (Howie et al., 2009). The most popular ones are FImpute (Sargolzaei et al., 2014) and Beagle (Browning and Browning, 2013), which are based on Hidden Markov models (HMM) and assumptions derived from the Overlapping Sliding Windows (OSW) model, respectively. Numerous variables, including the quantity and makeup of members in the reference group, the size of the effective population, allele frequencies, and variations in the densities of the reference and imputed genotypes, affect the accuracy of imputation (Sargolzaei et al., 2014). The genotype's quality, which is assessed based on the call rate, is another important consideration. A genotype call rate is defined as the percentage of called SNPs among all the SNPs in a particular chip. When the call rate falls between 90 % and 95 % percent, a quality genotype is identified. For proper imputation to occur, accurate input genotypes are a prerequisite. Phasing and imputation errors in genotypes cause the offspring's genotype to deviate from the parental haplotypes (Klímová et al., 2020). Imputation accuracy is influenced by various factors. The size of the reference population of genotyped animals and the quantity of missing SNPs are important factors in addition to the quality of individual genotypes ((Klímová et al., 2020). The percentage of imputing success, expressed as the percentage of matches between the original and imputed SNP, is another precision metric (Carvalheiro et al., 2014). Imputation accuracy studies generally indicate that the size of the reference population matters; if it is large enough, the accuracy is high even though the percentage of missing markers is higher (Carvalheiro et al., 2014; Kranjčevičová et al., 2019). The need for a strong infrastructure to handle demanding computational tasks was highlighted by the steady rise in computational load, which was primarily caused by the quantity of input markers and samples as well as the size of the reference panels. There are now two free next-generation genotype imputation servers available: the TOPMed Imputation Server and the Michigan Imputation Server (Das et al., 2016). The user-friendly interface of the platforms allows users to input sample data (as a gzipped variant call format, one for each chromosome) and set desired parameters, such as selecting standard reference panels (e.g., 1000 Genomes Project phase 3, Haplotype Reference Consortium panel, TOPMed r2 panel), optional subpopulation, quality filtering to apply on the imputed data, and finally, deciding whether to perform imputation, phasing, or both procedures sequentially. For phasing and imputation, Michigan and the TOPMed Imputation Servers use Eagle 2.4 and Minimac4, respectively. When compared to other programs (like Beagle 5), the combination of Eagle 2.4 and Minimac4 was found to be among the slowest methods; however, it was also the most resource-efficient for all input sample sizes (Browning et al., 2018), indicating the best option for online queued systems (Treccani et al., 2023).

### 4.6 Harnessing low-pass sequencing and genotype-by-sequencing for efficient imputation in GWAS studies

Low-pass sequencing or genotype-by-sequencing methods are becoming more and more popular (Elshire et al., 2011). Whole-genome sequencing at a shallow depth (i.e., low-pass or skim sequencing, 0.5 ×) can allow much denser genomic data at a fraction of the cost of deeper sequence when combined with imputation. It is not novel to generate lower-density data along with imputation; it is similar to genotyping using low-density assays and imputing to higher (e.g., 50,000 SNP) material. The attraction of low-pass sequencing solutions is twofold: more variants, including potentially causative mutations, and the ability to alter the set of variants actually fitted in genetic evaluations without having to redesign a custom assay, making the process of including new variants more dynamic. This is true even though SNP arrays of sufficient density are in use at a price point that has encouraged wide-spread genotyping throughout many livestock species. Nevertheless, this approach does have two main drawbacks: firstly, it requires a reference population that is representative, and secondly, it may not accurately determine genotype calls for significant variants as effectively as imputation (Berry and Spangler, 2023). An exemplar reference collection of animals with deeper sequencing depth (e.g., 10 ×) would ideally allow for high-accuracy imputation for every animal in the target population. Therefore, in order to guarantee that the reference can accurately represent the diversity of haplotypes in the current population, the reference set should initially include representative animals from any less connected sub-populations as well as modern high-use sires. A dynamic reference to time would be required. If imputation-mediated sequencing is not preferred, then targeted capture or genotype-by-sequencing methods, or higher sequencing depths that raise the cost of data generation, are required strategy would have to be used in order to produce genotypes (De Donato et al., 2013). Diagnostic markers, such as coat color, horn/poll, or genetic condition markers, may have significant variations. To ensure accurate genotype calls for such variants, targeted deep sequencing approaches are necessary. According to preliminary proof of concept research, this method can produce precise genotype calls (Snelling et al., 2020), and certain genotyping service providers appear to be leaning toward offering genotypes via low-pass sequencing-based products. Enhancing the accuracy of genetic merit prediction is the ultimate aim of utilizing imputed variants from sequence (Veerkamp et al., 2016; Warburton et al., 2020). Furthermore, higher-density genotypes might be required if the goal is to produce genomic assessments across strains or breeds (de Roos et al., 2008; Erbe et al., 2012). Ascertainment bias affects SNPs on SNP chips because the markers selected for the panel come from a sample population and are typically selected to be segregating in the breeds that are represented. However, sequencing techniques—whole-genome sequencing or genotype-by-sequencing approaches—can compensate for ascertainment bias (Berry and Spangler, 2023). By utilizing mitochondrial sequence produced by (low-pass) sequencing techniques, it is also feasible that this strategy could produce novel indicators, or bio-markers, of characteristics associated with efficiency (Sanglard et al., 2022). A list of recent research on the application of imputation methods in cattle genomics is reported in Table 5.

**Table 5.** A list of recent research on the application of imputation methods in cattle genomics.

Study	Used chip	Breed	No. of animals	Objective
Kamprasert et al. (2024)	Medium-density (50 K)	Angus	396 + 2380	Examining the effect of a within-breed and multi-breed reference panels on genotype imputation in Angus beef cattle
Sousa et al. (2024)	Medium-density SNP panel	North American Holstein	25,580	To perform a GWAS for 8 hoof-related traits and a hoof health index in Canadian Holstein cattle using imputed HD SNP genotypes
Korkuć et al. (2023)	Illumina BovineSNP50 chips, custom DSN200K SNP chips, and whole-genome sequencing	German Black Pied	2160	To fine-map genomic loci for milk production traits and to provide sequence variants for selection in German Black Pied cattle
Ogawa et al. (2023)	Illumina BovineSNP50 v2 BeadChip	Japanese Black	575	Assessing the performance of MCA and MCG methods of genotype imputation to identify the best animals for dense genotyping to construct a reference population
Oliveira et al. (2023)	Illumina BovineHD Bead Chip (HD-777 K), BovineSNP50 Bead Chip v2 (50 K), GGP Indicus (34 K), Z-Chip (30 K), and GGP Indicus (50 K)	Gir	22,216	To establish a platform for genomic selection of in vitro-fertilized (IVF) Gir embryos
Zhao et al. (2023)	Illumina Bovine SNP50 BeadChip (50 K), GGP Bovine HD (80 K), GGP Bovine 100 K (100 K), GGP Bovine HDv3 (150 K), and Illumina Bovine HD BeadChip (777 K)	13 breeds	1095	Five machine learning methods for breed assignment and three methods for breed-informative SNP detection were compared with respect to various reference population sizes and numbers of most breed-informative SNPs using whole genome sequence data available for thirteen cattle breeds
Hayes et al. (2023)	Neogen 35 K or 50 K Beef SNP array	14 breeds	29,321	Carrying out multi-breed genomic analysis for tropical beef cattle in the absence of available pedigree data
Scott et al. (2023)	∼7 K to 50 K Bovine SNP arrays	Holstein, Jersey, and their crosses	139,898	Evaluating the potential impact of selection for the A2 milk allele on inbreeding and performance in Australian Holstein cattle
Sanchez et al. (2023)	Different versions of the 50 K SNP Beadchip	Six breeds	236,496	Assessing the importance of the X chromosome in the genetic determinism of 11 complex traits related to milk production, milk composition, mastitis resistance, fertility, and stature in dairy cattle
Id-Lahoucine et al. (2023)	Illumina BovineSNP50 BeadChip	Canadian Holstein	43,710	To investigate transmission ratio distortion using SNP-by-SNP and sliding windows approaches on Holstein cattle

## 5 Genome editing (GnEd)

One of the most recent technologies for genetic enhancement is GnEd. Animal breeders can now precisely target the addition, deletion, or replacement of base pairs in the genetic code to influence desired traits thanks to advanced biotechnology. GnEd specifically describes the application of site-directed nucleases (i.e., cleaving enzymes for nucleic acids) to precisely introduce double strand breaks (DSB) at specific genomic locations in the DNA (Gaj et al., 2013). The application of genome editing technologies has been observed in various livestock production domains, including disease-resistant animal breeding, animal performance enhancement, milk composition enrichment, and hornless animal production (Alberio and Wolf, 2021; Koloskova et al., 2021). Furthermore, gene deletion for therapeutic and medical research purposes is a common application of CRISPR (Butler et al., 2016). Animal genome editing is currently experiencing a scientific revolution thanks to the CRISPR/Cas system (Wiedenheft et al., 2012). Tools for genome editing have numerous applications that can raise livestock productivity and related industries' profitability. Gene knockout and knockdown methods were previously used for gene editing in livestock, but they were ineffective and challenging to use, partly due to the scarcity of germline embryonic stem cells (Oishi et al., 2016; Raza et al., 2022). Moreover, since the majority of livestock attributes that are significant economically are quantitative in nature (i.e. regulated by numerous genes), improving cattle through genetic engineering nearly invariably necessitates editing the genome at several locations, which is excessively difficult and costly when employing conventional gene editing techniques. Therefore, alternative gene editing technologies that are able to precisely and efficiently edit multiple gene loci scattered across the genome of any host species are required if there is to be any real translational impact in terms of enhanced productivity and profitability of livestock production (Raza et al., 2022). Zinc finger nucleases (ZFNs), transcription activator-like endonucleases (TALENs), CRISPR/Cas9, and meganucleases are the four main categories of programmed nuclease-based technologies. The application of these genome editing tools has resulted in increased productivity, allergy-free living, climate response, and disease resistance (Gim et al., 2022, 2023; Lee et al., 2020). The U.S. Food and Drug Administration (FDA) in the United States has announced that genome-edited cattle will be permitted as low-risk (Van Eenennaam et al., 2019). It is anticipated that the development of gene-editing in cattle will accelerate in response to these changes in order to improve the traits.

### 5.1 GnEd tools

#### 5.1.1 Meganucleases

Endonucleases known as meganucleases are able to selectively target and cleave relatively long DNA sequences (14–40 bp) in vitro and/or in vivo. Even in the presence of polymorphisms, meganucleases can effectively bind and cleave these target sequences because their recognition sequences are rather long.

#### 5.1.2 ZFNs

Numerous meganuclease families have been thoroughly investigated, most notably the LAGLIDADG protein family, which shares the LAGLIDADG motif essential for the enzymes' activity. There are no recognition or cleavage sequences for naturally occurring meganucleases in the genomes of livestock. Therefore, the use of these enzymes in livestock is complicated because associated recognition sites must first be introduced by transfection into target loci if meganucleases are to be used for gene manipulations in livestock. Another class of proteins that have motifs that can bind to particular DNA sequences are called ZFNs. Zinc finger nuclease domains are structurally compacted due to the binding of two histidine and two cysteine amino acid residues to Zn^2+^. Through the a-helix residues, the ZF motif attaches itself to the major groove of the DNA double helix (Pavletich and Pabo, 1991). Several zinc fingers can join together to create a DNA recognition domain with increased specificity. ZFNs have a non-specific *Fok1*endonuclease cleavage domain in addition to a specific DNA binding domain. Certain genomic modifications typically require a pair of ZFN motifs.

#### 5.1.3 TALENs

Certain genome editing techniques make use of TALENs, which are naturally occurring virulence factors that were first discovered in the rice-infecting pathogenic bacterium Xanthomonasoryzae (Boch and Bonas, 2010). The TALENs system has been used to alter DNA binding domains in livestock in order to identify specific endogenic sequences. TALENs can activate HR and NHEJ mechanisms by affixing their binding domains to nonspecific cleavage domains from type II restriction endonucleases Fok1 (Miller et al., 2011). By cleaving DNA and causing non-homologous end-joining (NHEJ), TALEN can effectively be used to modify genes in livestock (Hockemeyer et al., 2011) leading to genetic alterations in various animals, including cattle (Proudfoot et al., 2015). Notably, target DNA with a thymidine nucleoside at the beginning of binding sites is typically recognized by a combination of TALEN repeats (Boch and Bonas, 2010). When screening possible target sites, this is a crucial factor to take into account. TALENs have numerous benefits over ZFNs: 1) The TALEN repeat is three to four times longer than the ZFN repeat. TALENs are more sophisticated than ZFNs because they only recognize one nucleotide, whereas ZFNs recognize three, 2) Because of the possibility of crosstalk between the fingers preventing successful DNA recognition, the ZFN modification necessitates a high-level design, 3) Because TALENs are simpler and can be produced more quickly and cheaply than ZFNs, they are easier to design, 4) Compared to ZFNs, TALENs have fewer off-target effects, and 5) When TALENs are injected into the cytoplasm of livestock embryos, they are more amenable to genome-editing than ZFNs (Gim and Jang, 2024; Khan et al., 2019).

#### 5.1.4 CRISPR/Cas9

In contrast to other mammal species, cattle genome editing hasn't advanced as quickly because of a few problems like lengthy gestational periods, single pregnancies, and high costs. Additionally, very skilled individuals using somatic cell nuclear transfer (SCNT) and microinjection technologies are required to produce the genetically modified cattle that have been produced to date. In particular, aberrant reprogramming problems like embryonic absorption and abrupt death allow us to be difficult to advance even though SCNT with high frequency mutated somatic cells have contributed to similar embryonic developmental competence with *in vitro* fertilized embryos (Gouveia et al., 2020; Thuan et al., 2010). Consequently, far more surrogate dams are required to generate a live cloned offspring than with the combination of *in vitro* fertilization and microinjection. Additionally, some research has shown that electroporation, a simple and effective substitute for microinjection, can be used to knockout animals (Camargo et al., 2020; Chen et al., 2016; Gim et al., 2022, 2023). Although there are many different genome editing technologies available, CRISPR/Cas9 based technologies are significantly better than other technologies because they are simple to use, effective, quick, and affordable (Raza et al., 2022). Characterizing a gene's function in relation to the pathophysiology of disease and the host immune response has become possible thanks to the widespread use of genome editing tools in recent years. It's also important to note that while most recent efforts utilizing CRISPR/Cas9 technologies focus on gene coding regions, these technologies can also target noncoding regulatory regions of the genome, for example promoters and enhancers. Additionally, genome-wide association studies can be combined with CRISPR/Cas9 technologies to functionally characterize markers for economically significant livestock traits. Under these conditions, nucleotide substitutions or targeted insertions/deletions can be performed using CRISPR/Cas9 technology to either knockout genes or alter regulatory elements that affect gene expression (Petersen, 2017; Raza et al., 2022). The CRISPR/Cas technique faces many obstacles, but remarkable progress has been made in the last few years that will open up the possibility of developing sustainable disease control strategies for livestock improvement—a difficult and time-consuming process (Raza et al., 2022).

### 5.2 Utilizing GnEd for targeted trait enhancement and environmental adaptation in cattle breeding

When it comes to introducing beneficial traits from different species or even useful genetic variation from one breed of cattle to another without undesirable linkage drag, GnEd present promising opportunities in cattle breeding programs. GnEd research in cattle has been centered on improving monogenic, or Mendelian, traits, and it is a suitable approach. Most Mendelian traits, like coat color or horn/pollled characteristics, are qualitative in nature and are governed by one or a small number of loci with significant effects. Nonetheless, a small number of identified single genes have significant effects on significant quantitative traits. For instance, a naturally occurring mutation in the myostatin (MSTN) gene causes a significant increase in the quantitative trait known as muscle yield, which is found in some cattle breeds such as the Belgian Blue (Kambadur et al., 1997; McPherron and Lee, 1997; Mueller and Van Eenennaam, 2022). GnEd has the ability to enhance genetic variation of a trait in the population, thereby increasing the rate of genetic gain, if it is used to target a gene that has a significant impact on a quantitative trait, such as MSTN. It should be mentioned that full knockouts of MSTN have also led to higher birth weights, which can result in dystocia problems, so more accurate MSTN mutations will probably be needed before this target can be used in real-world applications (Proudfoot et al., 2015). Nonetheless, the majority of the characters that breeders aim to enhance in animals are quantitative and polygenic (e.g., growth, feed efficiency, marbling, etc.). Quantitative genetics and genomic selection have been, and will remain, the primary forces behind genetic advancement for these traits. Furthermore, GnEd in cattle can only occur with the application of assisted reproductive technologies. In order to accelerate genetic gain by simultaneously changing several aspects of the breeder's equation, GnEd can only be fully utilized in conjunction with assisted reproductive technologies and genomic selection in a structured breeding program with a clear breeding objective (Bishop and Van Eenennaam, 2020; Mueller and Van Eenennaam, 2022). Gene editing technologies can generally be used to lessen the detrimental effects of heat stress on cattle productivity. It is possible to introduce mutations linked to heat tolerance into breeds that are thermosensitive; likewise, it is possible to introduce mutations linked to milk yield and composition into breeds that are thermotolerant but have low productivity (like native breeds). Spem from non-adapted bulls with high genomic value can be transferred by surrogate sires from adapted breeds to facilitate natural mating. Altering the expression of genes in Archaea through gene editing could also be used to control the amount of methane produced in the rumen (Camargo et al., 2023). A summary of recent literature on genome editing studies in cattle is shown in Table 6.

**Table 6.** A summary of recent research on genome editing in cattle.

Study	Goal/Targeted trait(s)	Genome editor	Major results
Wei et al. (2023)	Production of light-coloured, low heat-absorbing Holstein Friesian cattle	TALEN	Precision edited genotypes of multiple non-mosaic calves, including calves from parents with high genetic merit, were produced. Lower thermal energy absorbance was correlated with the edited calves' strong coat color dilution.
Gim et al. (2023)	Generation of double knockout cattle via CRISPR‑Cas9 ribonucleoprotein (RNP) electroporation	CRISPR/Cas9	Newborns with the *PRNP* and *MSTN* mutations in beef cattle and the *BLG* and *MSTN* mutations in dairy cattle are now available.
Bruter et al. (2023)	Editing *CD209* and *BLG* genes in cattle	CRISPR/Cas9	The necessary genome modifications were discovered in 5 out of 17 embryos that resulted from microinjections of guide RNA against the *BLG* gene and SpCas9 mRNA, and in 2 out of 9 embryos that followed microinjections of guide RNA against the *CD209* gene and SpCas9 mRNA.
Shaizadinova et al. (2023)	Identification of bovine mastitis milk samples contaminated by *Escherichia coli*	CRISPR/Cas12a	Using a novel MbCas12a nuclease, it was possible to identify *E. coli* isolates from milk samples from cows that had mastitis with excellent diagnostic performance.
Liao et al. (2022)	Diagnosing lumpy skin disease in cattle	CRISPR/Cas12a	For the purpose of quickly, accurately, portable, and highly adjustable lumpy skin disease diagnosis in cattle, a novel diagnostic tool was made available.
Wang et al. (2022)	Obtaining polled Holstein fetal bovine through inserting the Polled Celtic (Pc) mutation locus	CRISPR/Cas9	A theoretical framework and concepts for investigating the mechanisms of horn development and Pc locus regulation in Holstein cattle were presented. In order to increase the security and financial effectiveness of breeding cattle on farms, techniques for producing polled Holstein bulls were also proposed.
Zhao et al. (2022)	The myostatin gene (*MSTN*), a negative regulator of skeletal muscle development, was knocked out in Chinese Yellow Cattle	CRISPR/Cas9	The Chinese Yellow cattle with modified growth characteristics and normal fertility due to the *MSTN* gene editing can be utilized for breeding and production of beef cattle.
Laible et al. (2021)	Lightening the coat color of Holstein Friesian cattle by genome editing to better adapt dairy cattle to rapidly warming climates	CRISPR/Cas9	For the first time, it was confirmed that the PMEL mutation is the cause of the diluting of the black coat color in cattle.
Silaeva et al. (2020)	Knockouting *BLG* gene in cows for producing cows with hypoallergenic milk	CRISPR/Cas9	To create cattle that lack the *BLG* gene, a CRISPR/Cas9 system was developed to edit the cattle genome. It was anticipated that the offspring animals' milk would not contain *BLG* protein.
Schuster et al. (2020)	Producing a polled genotype of dairy cattle by knocking-in of the Polled Celtic variant	CRISPR/Cas12a	The CRISPR/Cas12a system was developed as a cutting-edge technique to insert the Pc variant into the superior Holstein–Friesian bull's genome.

## 6 Conclusions

The sustainability of animal agriculture has been and will continue to be significantly influenced by the global genetic improvement of cattle. Genetic improvement programs for cattle, starting with statistical prediction methods-based selective breeding (e.g., estimated breeding values) and more recently, genomic selection, in conjunction with assisted reproductive technologies have made it possible to accelerate rates of genetic gain by enabling more precise selection and intensive use of genetically superior parents for the following generation. By controlling the inbreeding coefficient, shortening the generational interval, and improving progenitor selection, the use of genomic analysis in cattle selection programs yields very intriguing predictions for the genetic improvement in the short- to medium-term, leading to the production of more productive genetic lines. The use of GWAS has expanded along with the development of high-throughput sequencing technology, computer software, and algorithms. These strategies' computing efficiency has also continued to rise. But the GWAS bottleneck has always been the identification of causal variation, which post-GWAS techniques may help to overcome. The most current development is the capacity to employ GnEd to deactivate specific gene function (i.e. knock-outing genes) presents encouraging chances to incorporate beneficial genetic variation into cattle breeding programs through knockout genes, knock-in genes, or allele introgression in the absence of unwanted linkage drag. Three main areas of improvement—animal health and welfare, product yield or quality, and reproduction or innovative breeding schemes—have received the majority of attention in GnEd experiments conducted on cattle. These areas are all closely related to the objectives of traditional breeding programs. At the moment, GnEd works well for introgressing alleles that typically affect qualitative Mendelian traits at a faster rate than can be achieved with traditional selection alone. Nonetheless, the majority of the characters that breeders aim to enhance in animals are quantitative and polygenic. Furthermore, the only way to achieve GnEd in livestock is by using assisted reproductive technologies. As a result, for GnEd to be a useful tool for genetic modification, it must be easily incorporated into a well-defined breeding program with a clear breeding goal. Moreover, it should ideally be used in tandem with genomic selection and assisted reproductive technologies to maximize genetic gain by simultaneously changing several aspects of the breeder's equation.

## Availability of data and materials

No data is associated with this manuscript.

## Consent for publication

Not applicable.

## Ethical approval

This article does not contain any studies with human participants performed by any of the authors.

## CRediT authorship contribution statement

**Navid Ghavi Hossein-Zadeh:** Writing – review & editing, Writing – original draft, Visualization, Validation, Supervision, Resources, Project administration, Investigation, Conceptualization.

## Declaration of competing interest

The authors declare that they have no known competing financial interests or personal relationships that could have appeared to influence the work reported in this paper.
